# Metabolite Changes during Postharvest Storage: Effects on Fruit Quality Traits

**DOI:** 10.3390/metabo10050187

**Published:** 2020-05-08

**Authors:** Delphine M. Pott, José G. Vallarino, Sonia Osorio

**Affiliations:** Departamento de Biología Molecular y Bioquímica, Instituto de Hortofruticultura Subtropical y Mediterránea “La Mayora”, Universidad de Málaga-Consejo Superior de Investigaciones Científicas, Campus de Teatinos, 29071 Málaga, Spain; dpott@uma.es

**Keywords:** fruit, postharvest, metabolomics, quality traits, stress, biomarkers

## Abstract

Metabolic changes occurring in ripe or senescent fruits during postharvest storage lead to a general deterioration in quality attributes, including decreased flavor and ‘off-aroma’ compound generation. As a consequence, measures to reduce economic losses have to be taken by the fruit industry and have mostly consisted of storage at cold temperatures and the use of controlled atmospheres or ripening inhibitors. However, the biochemical pathways and molecular mechanisms underlying fruit senescence in commercial storage conditions are still poorly understood. In this sense, metabolomic platforms, enabling the profiling of key metabolites responsible for organoleptic and health-promoting traits, such as volatiles, sugars, acids, polyphenols and carotenoids, can be a powerful tool for further understanding the biochemical basis of postharvest physiology and have the potential to play a critical role in the identification of the pathways affected by fruit senescence. Here, we provide an overview of the metabolic changes during postharvest storage, with special attention to key metabolites related to fruit quality. The potential use of metabolomic approaches to yield metabolic markers useful for chemical phenotyping or even storage and marketing decisions is highlighted.

## 1. Introduction

Fruit growth, ripening and senescence are complex processes, controlled by multiple developmental and environmental signals, and their molecular mechanisms remain unclear [1]. Fruits undergo important metabolic changes during ripening, including chlorophyll breakdown, anthocyanin or carotenoid pigment accumulation, cell wall degradation and the synthesis of low-weight metabolites (such as sugars, acids and volatiles), which function to increase their attractiveness to seed dispersers [2]. Once fruits are removed from the plant and until they reach consumers on the market, a period known as postharvest ripening or senescence occurs—the duration of which is variable (from days to weeks) and the effects of which mainly depend on fruit metabolism and ripening status at harvest. Indeed, climacteric fruits, such as tomatoes, kiwi or avocados, which exhibit a concomitant peak of ethylene production and a sudden rise in respiration at the onset of ripening [3], can ripen after harvest. In this sense, the control of ethylene production is fundamental to optimize the postharvest storage of these types of fruits [4]. On the other hand, non-climacteric fruits, e.g., strawberries and grapes, do not exhibit respiration and ethylene production peaks, and have to be harvested (almost) fully ripe. Postharvest storage initiates fruit senescence—the effects of which on biological processes are unavoidable and largely negative. Senescence leads to protein, lipid and nucleic acid degradation and cell dysfunction, disintegration and death [5]. Several factors influence and accelerate fruit senescence, with the most relevant being respiration, providing energy for maintaining metabolism, dehydration and fungal activity [6]. Consequently, the degradative processes associated with postharvest senescence impact fruit quality traits, i.e., aspect, texture, taste, aroma and nutritional characteristics, leading to consumer rejection and important economic losses for the fruit industry.

Currently, depending on fruit crops, different postharvest strategies are commercially practiced in order to adapt ripening to industry needs, delay senescence, maintain fruit quality attributes and, thus, prolong shelf-life. In general, fruits are highly perishable at ambient temperature. Thus, refrigerated storage is the most common method used to delay ripening, fruit respiration, enzymatic activities, and the development of pathogen infections, and, therefore, extend fruit shelf-life [7]. However, cold storage can provoke the development of a physiological disorder called chilling injury (CI). Although CI symptoms are species dependent, CI includes internal and external browning, mealiness, flesh bleeding, pitting or the inability to soften. These physiological disorders tend to appear once the fruits are acquired by consumers, having a negative impact on palatability and acceptance [8,9]. To reduce CI symptoms and depending on the type of fruit, the industry combines low-temperature storage with some complementary strategies. For example, prior heat treatment to cold storage is widely used in several crops, including *Citrus* and loquat [10], while controlled atmosphere (CA, increased CO_2_ and decreased O_2_ levels) is commonly applied to apple, strawberry, peach and pear, among others [11]. Additionally, delayed cooling has been successfully applied in apple to reduce soft scald, a chilling-dependent physiological disorder [12]. While CA reduces fruit respiration, heat treatment has a protective effect by acting on membrane integrity and heat shock protein accumulation and by promoting antioxidant and sugar metabolism [13]. Further, it is known that heat treatment induces defense mechanisms and induces physiological changes that allow *Citrus* fruit to withstand stressful conditions during storage. For example, GC–MS analysis in heat-treated oranges during storage showed a higher concentration of sugars while no changes were observed in organic acid levels [14].

In the case of climacteric fruits, such as tomato or banana, the application of ethylene antagonist 1-methylcyclopropene (1-MCP) is commonly used to increase shelf-life [15]. However, the aforementioned strategies have different degrees of effectiveness at reducing CI and prolonging shelf-life, depending on fruit species and varieties. In addition, it must be highlighted that these postharvest techniques constitute abiotic stresses for the fruits, which have to adapt their metabolism to maintain homeostasis [16]. In particular, stress situations induce the synthesis of compounds involved in plant protection, and trigger the accumulation of compatible metabolites, reactive oxygen species (ROS)-scavenging enzymes and changes in carbon metabolism [17,18]. In this sense, metabolomic platforms, allowing the simultaneous detection and quantification of hundreds of metabolites, offer the possibility to improve our knowledge about the molecular mechanisms underlying fruit senescence under commercial storage conditions.

## 2. Metabolomic Platforms in Postharvest Studies

The plant metabolome comprises a wide range of small molecules, with a large variety in physico-chemical properties and extremely variable concentrations. Metabolomics is defined as the field of the research that generates a profile of small molecules in a biological system. Thus, it can directly reflect the outcome of complex networks of biochemical reactions and, therefore, provides essential information about the underlying biological status on the system in question.

For these reasons, successful analysis of the complex network of fruit metabolites requires highly sensitive and selective analytical techniques, with each displaying both advantages and limitations and showing differential coverage depending on the nature of the metabolite. In particular, mass spectrometry (MS) coupled with gas chromatography (GC), liquid chromatography (LC) and, to a lesser extent, capillary chromatography (CE) and nuclear magnetic resonance spectroscopy (NMR) have been the most extensively applied methodologies to study the plant metabolome, including its reconfiguration during fruit postharvest senescence [17,19,20].

GC–MS is the technique of choice for measuring small polar metabolites, which are thermally stable and can be made volatile through a derivatization approach [21]. The main advantages of GC–MS are its robustness and reproducibility, which have allowed the establishment of libraries and databases facilitating the identification of metabolites. As a result of its characteristics, GC–MS is mainly used in plant metabolomic studies to investigate central primary metabolism, which includes sugars, sugar alcohols, amino acids, organic acids and polyamines [17,19]. In addition, GC–MS can be coupled with headspace solid-phase microextraction (HS-SPME), which allows the detection of specific volatiles present in a sample [22]. Both volatile and primary metabolite changes occurring during fruit postharvest storage have been extensively studied, as they are key compounds of fruit taste and aroma.

To overcome the limitations of GC–MS, which is restricted to volatile and thermally stable molecules, LC–MS is broadly used to detect a wider range of metabolites. In particular, the enormous diversity of plant secondary metabolites, which includes tens of thousands of different compounds [23], is mainly studied using LC–MS due to its versatility. However, and as a consequence of LC–MS flexibility, metabolite identification remains difficult, as no universal mass spectral library has been created [24].

Another technique used to study plant metabolomics, although rather uncommon, is capillary electrophoresis (CE)–MS. This technique allows the detection of a wide range of highly polar or charged metabolites by separating them based on their mass-to-charge ratio [25]. In this sense, this method has been proposed as a valuable complementary approach for samples that cannot be readily resolved by the more established GC– and LC–MS platforms [26].

Even if NMR presents a low sensitivity compared to that of MS approaches, it provides a series of advantages over the previously mentioned approaches by providing structural information, involving non-destructive sample preparation and providing rapid metabolite screening [19,27]. Integrated NMR platforms, allowing the monitoring of changes in both primary and secondary metabolites, have been developed and can be useful to study metabolic shifts in senescent fruits during postharvest [28].

## 3. Primary Metabolic Pathways Affected by Postharvest Storage: Effects on Fruit Texture and Taste

Fruit organoleptic quality is a complex trait that is influenced by taste, aroma, color and texture. In particular, fruit acceptance by consumers is directly influenced by sugar and acid content and the ratios of both groups of primary metabolites [29,30]. Fruit respiration during postharvest storage directly affects primary metabolic pathways, such as glycolysis, starch metabolism, and the tricarboxylic acid cycle (TCA), which account for changes in sugar, amino and organic acid levels. Indeed, carbohydrates, organic acids, proteins and fats are the main respiratory substrates during fruit storage. Furthermore, they are involved in gluconeogenesis, a process which has been described to be upregulated during the postharvest in orange and apple fruits [31,32,33]. Thus, it contributes to fruit depletion and also to important changes in primary metabolite composition. In the next paragraphs, we described alterations in sugars, organic and amino acids as a consequence of postharvest storage.

### 3.1. Sugars and Sugar Derivatives

Sugar content, which is commonly estimated by the soluble solid content (SSC), shows differential behavior during postharvest, depending mainly on the species and storage conditions. The SSC trend normally coincides with changes in the main sugar profiles present in ripe fruits, i.e., glucose, fructose and sucrose.

While main carbohydrates tend to decrease in some species, as profiled by GC–MS and NMR analysis in tomatoes kept at room temperature [34] or blackberries stored at 4 °C [35], in other fruits, such as bananas and kiwis, their level increases as a consequence of starch hydrolysis, which takes place during postharvest storage [36,37]. In turn, sucrose can be hydrolyzed, leading to a concomitant increase in hexoses, as monitored by GC–MS in Powell oranges stored at room temperature [33]. Interestingly, growing evidence seems to point to sugars playing a regulatory role in senescence processes [38,39]. During fruit ripening and senescence, cross-talk between sugars and hormones involved in ripening and senescence processes, such as abscisic acid, ethylene and auxin, has been described [40,41,42,43], and sucrose degradation during postharvest storage can be crucial for inducing senescence [33,44]. Furthermore, sugar uptake during fruit ripening may affect postharvest water loss by interfering with cuticle development. Indeed, stable silencing of the cell wall invertase *LIN5*, a key determinant of SSC content, led to a diminished water loss rate and wrinkling in transgenic tomato fruits kept at room temperature for 12 days. Even though the complete molecular mechanism has not been described, it was clearly established that sugar entry during fruit development impacts the cell wall and cuticle structure, resulting in a radical effect on tomato senescence [45].

Apart from the most abundant sugars, i.e., sucrose, glucose and fructose, fruits also contain minor sugar and alcohol derivatives, such as sorbitol, galactinol, raffinose, *myo*-inositol and trehalose [46]. Even if those compounds may be at low concentrations, they seem to be crucial for fruit behavior during storage, as they can alleviate the negative effects of the abiotic stresses underlying postharvest conditions. Indeed, soluble sugars are important metabolites in ROS metabolism, being the primary carbon and energy source and contributing to the generation of reducing power generation via the oxidative pentose phosphate pathway [41,47,48]. Furthermore, they play key roles in osmoprotection and cell membrane stabilization [49,50,51]. As an example, important increases in raffinose and galactinol levels were measured by GC–MS in peaches after heat treatment (three days at 39 °C) followed by storage at 0 °C for two days and may confer improved tolerance to CI [52]. Moreover, comparing the levels of galactinol (detected by LC–MS/MS), raffinose, trehalose and *myo*-inositol (identified by NMR) in climacteric and non-climacteric plum varieties during postharvest storage at 20 °C and in presence of 1-MCP, propylene (ethylene analogue) or control air, Farcuh et al. [46] noticed that the levels were more enhanced in the latter variety. These data could explain the capacity of the non-climacteric variety to cope better with postharvest stress conditions, and the identified sugars could be used as biomarkers to evaluate fruit physiological status during storage (Table 1).

Softening during postharvest storage is a key physiological process leading to ripe fruit firmness; however, excessive loss of firmness as a consequence of overripening can prompt physical damage and pathogen attack, and consequently lead to an important decrease in fruit quality. Softening is the result of several factors, including cell wall disassembling metabolism. Metabolites originated from cell wall disassembly, mainly monosaccharides, can be monitored by primary metabolite profiling. Indeed, in pitaya fruit, the content of several monosaccharides, including xylose, galactose, arabinose, and mannonic acid and glucuronic acid, which originate from cell wall disassembly, was measured by GC–MS [53]. Interestingly, these metabolites were decreased after blue light treatment (2 h at 25 °C under blue light emitting diode) compared to control fruits kept in the dark, suggesting that this treatment has a significant effect in delaying cell wall degradation and postharvest decay of pitaya fruit [53]. Another study, using LC coupled with tandem MS (LC–MS/MS), detected an increase in glucuronic acid, a component of pectin, among the major elements of plant cell wall, in pears stored 18 days at room temperature [54]. Pectin de-polymerization and de-esterification were also evidenced by the detection of galacturonic acid by two-dimensional GC–MS (GC x GC–MS) in overripe kiwi fruits stored at 20 °C and treated for 24 h with 200 ppm ethylene. On the contrary, no increase in galactose was observed using GC–MS measurements, suggesting that this sugar is directly metabolized after its release from cell wall, or that it is liberated as different form [55]. Among the main symptoms of CI in peach is mealiness, which is the result of a cell wall metabolism disorder. Xylose, the central constituent of hemicellulose, among the key components of plant cell wall, was increased during cold storage in peach chilling-susceptible genotypes, but not in the varieties resistant to CI, confirming a link between cell wall disassembly and mealiness in sensitive cultivars [56].

### 3.2. Organic Acids

Several organic acids are related to fruit postharvest metabolism. Surprisingly, particularly in tomato, the levels of malate, among the most abundant organic acids, impact fruit shelf-life (Table 1). The malate content, measured by GC–MS, decreases during the ripening and postharvest storage at room temperature of several tomato genotypes, including *delayed fruit deterioration*, *non-ripening* and *ripening inhibitor* mutants as well as genotypes that are commercially used because of their delayed maturation and senescence. Interestingly, it was also shown that malate levels were lower in mature tomato fruits that ripened on the vine than off the vine [34]. However, when the malate concentration in tomato fruits was manipulated by reducing the expression of two TCA cycle enzymes (*fumarase* and *malate dehydrogenase* (*MDH*)), a differential postharvest behavior was observed compared to that in wild-type fruits at room temperature. Interestingly, fruits of the MDH-deficient genotype showed higher malate content and poorer postharvest behavior than non-transformed fruits by losing more water and being more susceptible to opportunistic fungal infections and *Botrytis cinerea* spores. In contrast, the fumarase-deficient genotype, with a relatively low malate content, presented a decrease in water loss and in susceptibility to opportunistic fungi [57]. The mechanism underlying malate’s role in postharvest responses could not be clearly explained; however, the authors suggested a role for SSC, which changes in the opposite manner in *MDH*- and *fumarase*-silenced lines, in osmotic potential and subsequent water loss during storage. Another study using recombinant inbred lines originating from the cultivated tomato *Solanum lycopersicum* and the wild-type species *Solanum pimpinellifolium* also pointed out the association between malate content, fruit firmness and shelf-life [58]. A comprehensive polar metabolite profiling was performed by GC–MS and NMR and a combination of neuronal clustering and network construction displayed a strong correlation between glycerate and malate content and postharvest, which also showed a negative correlation with fructose levels [58]. This association between metabolites and agronomic traits such as firmness and storage behavior suggested that malate could be a good biomarker to select genotypes with enhanced quality traits, such as improved postharvest life [58].

By performing a GC–MS metabolic characterization of *S. lycopersicum* cv. ‘Plaisance’ fruits during ripening and postharvest stages, Oms-Oliu et al. [59] showed that one sugar (mannose) and three organic acids (citramalate, gluconate and keto-gulonate) were strongly increased once the fruit was removed from the vine and that these compounds could be indicators of metabolic shifts during postharvest storage [59] (Table 1). As an example, the enhanced gluconate levels could be a consequence of tartarate biosynthesis from ascorbate degradation or energy balance changes during tomato storage [62,63]. Free mannose levels are generally low, as this monomer usually composes carbohydrate polymers. However, it can be found in a free form as a result of cell wall disassembly and hemicellulose breakdown during fruit senescence, as described in tomato, apple and pear [59,62,64].

Organic acids, particularly citric acid, accumulate at high levels in *Citrus* fruits, such as lemons, oranges, grapefruits or pummelos. A study on ‘Hirado Buntan’ pummelo focused on the relationship between organic acids, measured by high-performance capillary electrophoresis, and fruit senescence during postharvest storage at both ambient and cold temperatures. The authors observed a general decrease in malate, citrate, aconitate and fumarate during storage, accompanied by important fluctuations in their levels; this decrease was associated with a loss of fruit quality [31]. The combination of transcriptomic analysis paralleled the metabolomic data, suggesting that the *peroxisomal MDH*—the expression of which correlated with malate levels—is responsible for organic acid metabolism regulation during postharvest. This result indicated that the glyoxylate cycle, which occurs in peroxisomes and glyoxysomes, is central to organic acid regulation by supplying succinate for the TCA cycle [31]. Tang et al. [33] also observed a decrease in several organic acids analyzed by GC–MS, such as malate, citrate and α-ketoglutarate, during postharvest storage of ‘Powell’ oranges at room temperature. In this case, they suggested that malate could be used as a substrate for gluconeogenesis, being converted into phosphoenolpyruvate (PEP) by the action of two enzymes upregulated in oranges kept at room temperature: PEP carboxykinase and pyruvate orthophosphate dikinase (PPDK). Similarly, an increased abundance of PPDK proteins associated with decreased malate content was observed in peaches subjected to heat treatment followed by storage at 20 °C [65]. Another study using GC–MS analysis in different varieties from the *Citrus* genus suggested that a conversion of organic acids to sugars during fruit postharvest senescence at ambient temperature occurs, as negative correlations were frequently observed between metabolites belonging to the two groups and that the SSC/titratable acidity ratio increased during storage [66]. The succinate content increased during pummelo postharvest storage, showing a positive correlation with GABA and glutamine [31]. In addition, GABA increased during the postharvest senescence of Powell oranges, matched by an upregulation of the genes involved in the GABA shunt [33]. In this sense, the GABA shunt was outlined as an important pathway for organic acid catabolism and for balancing organic acid and amino acid levels. Indeed, superfluous citrate can be converted into amino acids via the GABA shunt [33,67]. Moreover, Sun et al. [31] observed an increase in ROS during pummelo storage, which correlated with enhanced mitochondrial damage. Cross-talk between ROS and organic acids could occur during postharvest senescence, as TCA enzymes have been described to be very sensitive to inhibition by ROS [68,69], while organic acids could be involved in the direct ROS scavenging [70,71].

### 3.3. Amino Acids

Amino acid content is also affected to a large extent by postharvest storage, as these compounds are involved in several pathways induced during fruit ripening [47]. In particular, during senescence, amino acid catabolism can counteract the reduction in electron supply from the TCA cycle [72]. Ubiquitination of proteins controls their degradation to free amino acids, and upregulation of the ubiquitin pathway has been reported in stored peaches that were previously were heat treated [73]. Dopamine, a derivative of the aromatic amino acid tyrosine, has been proposed as a postharvest marker in banana fruit stored at 25 °C [36] (Table 1). Indeed, NMR-based metabolite profiling of the senescence of bananas stored at room temperature showed that dopamine levels were undetectable at the last postharvest stage. Concomitant with dopamine disappearance was the sudden appearance of salsolinol, which has been described to originate from dopamine and acetaldehyde, the latter formed from ethanol, which is also generated in the late postharvest stage [36,74]. The authors concluded that the conversion of dopamine to salsolinol led to a decrease in fruit quality, making bananas less fit for consumption [36].

Additionally, several amino acids play a key role in tolerance to abiotic stresses in fruits during postharvest senescence. Indeed, a GC–MS comparative study between pineapple varieties tolerant and susceptible to CI stored at 10 °C outlined that amino acid increases during chilling stress may be associated with a delay in symptom appearance, such as internal browning, by presumably contributing to the synthesis of enzymes involved in tissue repair and, in the case of cysteine, aspartate and valine, by acting as osmoprotectants [75]. Proline is a well-documented stress-related amino acid and among the main osmolytes that are accumulated during plant stresses, playing important membrane protection and ROS-scavenging functions [76,77] (Table 1). Grape storage in a CO_2_-enriched atmosphere resulted in a threefold endogenous proline increase when compared to that in air-stored grapes [60], and proline accumulation is a common trend in postharvest fruits subjected to treatments to attenuate CI, such as zucchinis [61], mangoes [78], bananas [79], pears [80] and loquats [81]. However, the possible role of amino acids in counteracting CI seems to be species dependent, as GABA, aspartate, phenylalanine and proline increase in peach stored at 0 °C for 21 days was not associated with CI protection, since their levels, quantified by GC–MS, were enhanced in both resistant and susceptible genotypes [56].

A recent study in strawberry also outlined the possible role of amino acids in plant defense, as pathogen resistance mechanisms implicated this group of metabolites [82]. The increase in asparagine, aspartic acid, threonine, glutamic acid, glutamine, alanine and glycine in CO_2_-treated strawberries compared to control fruits could, at least partially, explain the lower fungal decay observed in the first group [83].

## 4. Postharvest Impact on Secondary Metabolites

The two main families of secondary metabolites present in fruits are polyphenol and terpenoid compounds, responsible for their appealing color and also important for their organoleptic and nutritional characteristics [84]. Apart from their importance in the human diet, these molecules are involved in plant defense and responses against biotic and abiotic stresses. In particular, metabolomic approaches have helped in deciphering their role during fruit storage and how different postharvest strategies impact on them. Here, impact on polyphenols, including anthocyanins, and carotenoids during fruit shelf-life is discussed in the next paragraphs.

### 4.1. Polyphenol Compounds

Dynamic metabolite changes, profiled by high-performance LC–MS (HPLC–MS), were observed during grape postharvest ripening and dehydration, the metabolic responses being genotype dependent [85]. A particular feature was the cultivar-specific accumulation of stilbenes, a class of phenylpropanoid compounds, with antifungal activity. On the other hand, anthocyanins and other flavonoids, belonging to another phenylpropanoid class, were depleted along postharvest dehydration [85]. An untargeted HPLC–MS profiling during grape ripening and withering (postharvest drying), combined with transcriptomic and proteomic data integration, also correlated the presence of stress-related secondary compounds (stilbenes and acylated anthocyanins) with the postharvest phase. The synthesis of defense molecules could be a response to abiotic stress (dehydration) or biotic stress (eventual pathogen attack). In addition, three metabolites (two taxifolins and tetrahydroxyflavanone-O-deoxyhexoside), belonging to the flavonoid class, have been proposed as putative markers in order to assess berry fruit quality traits (Table 2) [86]. In grapes, the accumulation of different stilbenes during cold postharvest storage was monitored by UHPLC–MS/MS [87]. This increase was also observed when grape fruits were kept at high CO_2_ [87]. In contrast, CA storage has been described to have negative effects on anthocyanin accumulation in strawberry fruits, compounds responsible for the color of the ripe fruit [88]. In this sense, postharvest cold storage is a mandatory strategy to enhance anthocyanin content in some fruits such as blood oranges, some varieties of plums and anthocyanin-rich tomatoes [89,90]. Interestingly, it has been described that tomato anthocyanin-rich lines are able to maintain fruit quality for longer during storage, mainly by reducing their susceptibility to *Botrytis cinerea* [91,92].

In mandarins, heat treatment previous to storage at 12–16 °C positively impacts polyphenol metabolism by increasing flavonoids and lignin content (flavonoids measured by HPLC–MS). The effect of this postharvest strategy can be seen as a modulation of fruit defense against biotic and abiotic stress during postharvest storage, by supplying chemical (flavonoids) and physical barriers against pathogen attack [93]. The relationship between polyphenol content and resistance to postharvest decay caused by *Penicillium expansum* has also been described in apple; indeed, resistant and susceptible apple genotypes could be discriminated based on polyphenol content, measured by UPLC–MS (Table 2) [94]. However, a general polyphenol increase during fruit shelf-life does not always occur, as described by untargeted UHPLC–MS in several mango varieties stored at room temperature during six days, in which it was found that only gallic acid and epicatechin content was enhanced after storage [95].

### 4.2. Carotenoids

Carotenoids are an important class of terpenoids, responsible for the attractive color of many fruits and vegetables. While their low stability during postharvest storage, mainly due to a rapid turnover of β-carotene, has been described in many staple crops, postharvest accumulation in *Citrus* and tomato seems to be temperature dependent [96,97]. Carotenoid levels in grapefruit, determined by HPLC, stored at 2 and 12 °C established a link between carotenoid content and CI symptom suppression, suggesting that they play a role in preventing cold damage by protecting plastid structures [98,99]. Furthermore, the ratio between 9-Z-violaxanthin (yellow hues) and β-citraurin (orange-red pigments), responsible for the external orange fruit color, was lower in sweet oranges stored at 12 °C than at 2 °C, outlining that this important quality indicator is better maintained at moderate temperatures. Additionally, increased levels of β-cryptoxanthin in orange pulp stored at 12 °C should be pointed out, due to health-beneficial provitamin A activity (Table 2) [96]. Carotenoid content, measured by HPLC, was also drastically increased during postharvest storage of winter squash at 21 °C, even if no induction of the biosynthetic genes could be observed. Starch degradation during winter squash storage, with the concomitant release of soluble sugars which may act as substrates for terpenoid synthesis, and downregulation of genes involved in carotenoid turnover, could be the explanation of their enhanced content [100]. In other fruits, such as green pepper, carotenoid accumulation during postharvest storage has a negative impact on consumer acceptance. Pepper reddening depends on the metabolic dynamic of chlorophyll degradation and active synthesis of carotenoids, such as β-carotene and capsanthin, as depicted by HPLC-based profiling of these pigments [101]. Quantification of chlorophyll by spectrophotometry has also pointed out its breakdown as a deterioration factor occurring during pear or lime shelf-life [102,103]. In this sense, postharvest strategies, such as chlorine dioxide fumigation or hot water treatment, may be effective in downregulating genes involved in chlorophyll-degrading enzymes [101,102].

## 5. Volatile Profiles during Postharvest and Their Impact on Fruit Aroma

In fruits, there are three major classes of metabolites responsible for flavor: sugars, acids, and volatile. While fruit taste is mostly dependent on the ratio of sugars and acids, it is the volatiles that determinate the unique flavor of fruits. Most volatiles present in mature fruits originate from terpenoid and phenylpropanoid pathways or are fatty and amino acid derivatives [104]. Volatile profiling is typically achieved by extracting them from the headspace (HS), i.e., the airspace around the fruit, and detecting them by GC–MS. Sampling from headspace is most often performed by the adsorption of the volatiles on a stationary phase coated on a fused silica fiber and is known as solid-phase microextraction (SPME) [104]. Another GC–MS-based strategy for volatile profiling is their collection from chopped fruits on a Super Q column, followed by elution with methylene chloride [105]. To overcome metabolite co-elution by one-dimensional GC, GC x GC–MS has been implemented to increase separation efficiency and volatile detection [106,107]. As not all volatiles impact fruit aroma, a complementary approach, known as GC—olfactometry, can be used to determine odor-active compounds [108]. During postharvest, it could be established that important shifts in fruit volatile profiles are normally observed and are often responsible for the decreased sensory acceptability after prolonged storage. For instance, general trends profiled by GC–olfactometry, describe a loss of ‘green’ or ‘fresh’ notes and a concomitant increase in ‘fruity’, ‘overripe’ or ‘musty’ aromas [109]. Changes in aroma are a consequence of metabolic pathways that are active during postharvest and, in turn, appear to be largely depend on the storage strategies used by industry. For example, among the symptoms related to CI is the negative impact perceived on aroma production, a phenomenon described in many species, such as strawberries [110], kiwifruit [111], tomatoes [112] and peaches [113]. Tomatoes stored at 5 °C for 7 days were significantly less palatable than fruits recently harvested, and this decrease in consumer acceptance, established by taste panels, was a consequence of changes in volatile emissions [105]. Furthermore, a higher increase in ‘musty’ and ‘damp’ aroma notes was observed in tomatoes stored at 10 °C than in those stored at 12.5 °C, suggesting that the latter temperature storage was able to maintain better sensory attributes [114].

Fermentation metabolism and amino acid and fatty acid catabolism are of great importance regarding the production and accumulation of volatiles in harvested fruits. Indeed, the activation of amino acid and fatty acid degradation to generate TCA cycle acetyl-CoA precursors and thus maintain energy production leads to the accumulation of specific substrates for volatile formation. In mandarin, a combination of metabolomic and transcriptomic data outlined the upregulation of genes involved in branched-chain amino acid catabolism, fatty acid cleavage and ethanol fermentation, which suggested that central metabolism modifications are accountable for the increase in branched-chain esters (‘fruity’, ‘overripe’ aroma), fatty acid-derived volatiles (‘musty’ notes) and ethanol [115]. The activation of anaerobic fermentative metabolism due to postharvest abiotic stress is especially important in ‘off-aroma’ compound generation and has been described in fruits of several species, including strawberries [116,117,118,119], apples [120], mandarins [109] and peaches [121], among others. Indeed, the glycolysis end-product pyruvate can alternatively serve as a substrate for anaerobic respiration and ATP production under O_2_-limiting conditions, which produces a shift from aerobic respiration to the fermentation pathway [16,120,122,123]. As a consequence, off-aroma volatiles, namely ethanol, acetaldehyde and ethyl acetate, accumulate, playing a key role in fruit quality decline [117,124] (Table 3).

Fermentative metabolism activation and off-flavor compound formation are mainly associated with low oxygen concentration under CA storage [118,123,124,131]. However, the production of ethanol via fermentation may also be a consequence of a decline in cellular energy status [132]. As long as energy demand is maintained, fermentation can be endured; nevertheless, failure of cellular homeostasis, such as an imbalance in the pH or ROS production, will lead to storage-induced disorders, strongly affecting fruit quality [133]. Understanding how or when fermentation occurs can help to limit ethanol production. Metabolomic approaches using ^1^H NMR and GC–MS profiling were used to assess metabolite gradients within the fruit, which may be related to in situ hypoxia in the central part of the ripening fruit [134,135]. CA is of special importance for the long-term storage of fruits such as apples, and could maintain a better aroma quality [136,137]. It appears that the low-oxygen pressure employed during CA affects volatile emissions in a genotype-dependent manner [11]. Indeed, a multiplatform metabolomic approach (proton-NMR, GC–MS and HS-SPME–GC–MS) comparing ‘Red Delicious’ and ‘Granny Smith’ apple varieties showed strong activation of fermentative metabolism in the former, with ethanol and acetaldehyde accumulation, while the latter dealt with hypoxia by a reconfiguration of nitrogen metabolism through the intensification of alanine levels to prevent excessive accumulation of pyruvate [11]. Low oxygen may induce changes in metabolite concentrations that reflect a decrease in biosynthetic process, inhibition of the TCA cycle, and activation of anaerobic metabolism, which means accumulation of sucrose and organic acids and diversion of pyruvate to ethanol and alanine [134]. Table grapes stored under elevated CO_2_ concentrations (5 kPa O_2_ and 15 kPa CO_2_) showed an upregulation of genes involved in pyruvate synthesis (*pyruvate kinase*, *PEP carboxykinase* and *NADP-dependent malic acid enzyme*) and a concomitant increase in volatiles, detected by HS-SPME–GC–MS, derived from pyruvate degradation—some of which were suspected to generate ‘off flavor’. Additionally, the increased expression of a specific *alcohol dehydrogenase* gene (*ADH*) under anaerobic atmospheric conditions enhanced the accumulation of off-aroma volatiles, including ethanol, acetaldehyde and ethyl acetate [87].

Metabolic reconfiguration during postharvest affects volatile patterns beyond the generation of off-aroma compounds, and changes occurring in most important volatile classes are described in the next sections.

### 5.1. Fatty and Amino Acid-Derived Volatiles

Fatty acid-derived volatiles, responsible for aldehyde, alcohol and ester accumulation, the last being the predominant class of aromatic compounds in fruits of several species, seem to be strongly impacted by low-temperature storage [110,138,139]. Free fatty acids such as linoleic acid and linolenic acid are reduced to aldehydes by the lipoxygenase pathway (LOX). Next, aldehydes are reduced to alcohols followed by alcohols to esters by ADH and alcohol acyltransferase (AAT), respectively (for a review, see [84]). Interestingly, correlations among LOX, ADH and AAT activities, gene expression and decreased volatile production under refrigerated postharvest conditions have been established in several fruit-bearing species [140,141]. In particular, a relationship between a reduction in ADH activity and decreased ester content, monitored by SPME–GC–MS technology, during pear cold storage has been established [138]. In tomatoes, ADH activity was diminished as a consequence of refrigerated conditions at both 10 and 12.5 °C, and storage was associated with an increase in the aldehyde/alcohol ratio at 10 °C [112,114]. Furthermore, a decrease in *ADH2*, *LoxC* and *AAT1* transcripts after 8 days of cold storage (5 °C) was associated with lower levels of C6 and C5 (fatty acid-derived) volatiles in chilled tomatoes [105]. Additionally, low temperature also seems to affect upstream lipid catabolism by downregulating the expression of several genes involved in the formation of the unsaturated free fatty acids linoleic acid and linolenic acid, limiting substrate availability for ester biosynthesis [142]. Furthermore, membrane damage during cold storage has also been suggested to impair ester synthesis, as a relatively high leakage rate, a commonly used marker for membrane permeability, was measured in pears stored for a long time under refrigerated conditions [138]. The impact of cold storage on the aromatic compound profile reaches further than that on the pattern of lipid-derived volatiles. Branched-chain volatiles derived from the direct precursors of branched-chain amino acids, measured by GC, after methylene chloride extraction, were also shown to decrease during tomato cold storage and are correlated with a lower expression of two branched-chain aminotransferases (*BCAT1* and *BCAT7*) involved in the first step of the catabolism of these amino acids [105]. Additional treatments, such as hot air or UV-C, combined with cold storage could counteract the negative effect on ester biosynthesis by promoting the *LOX* pathway, as has been demonstrated in peaches [143]. Similarly, a pre-chilling heat treatment (52 °C, 5 min) has been shown to alleviate the depletion of important volatiles for tomato aroma quality during its postharvest storage; in this case, the volatiles include amino acid- and carotenoid-derived compounds profiled by HS-SPME–GC–MS [144]. Fatty acid-derived alcohols were also higher under elevated CO_2_ concentrations compared to those of recently harvested grapes and cold-stored berries under atmospheric conditions due to the upregulation of the *LOX* pathway, together with *ADH* [87].

Low-oxygen storage has a broader impact on volatile content than ethanol and off-aroma compound generation, as demonstrated by the different content of ethyl esters between ‘Granny Smith’ and Red Delicious’ apples [11]. Indeed, ethanol can serve as a substrate for ethyl esters, enhancing their synthesis [136,145,146] and competitively inhibiting the formation of esters originated from other alcohols [147]. As a consequence, an imbalance between the ratio of ethyl and the remaining esters occurs during postharvest storage of fruits of ethanol-accumulating apple varieties and those of many other fruit-bearing species, most likely affecting aroma perception. The fruits of ‘Granny Smith’ and ‘Royal Gala’ apple varieties did not seem to accumulate ethanol under low-oxygen-pressure storage; however, a negative effect on ester synthesis, in particular straight-chain esters, was observed, with the impact proportional to the decrease in O_2_ pressure [148,149,150,151,152,153,154]. This decrease can be explained by the fact that the LOX pathway requires the presence of oxygen. This effect has also been described in other apple varieties. [137,153]. In contrast, the concentration of branched-chain esters, monitored by HS-SPME–GC–MS, did not seem to be negatively affected by low oxygen, possibly because branched-chain amino acid levels were unaltered [154]. Furthermore, low oxygen suppresses the production of the hormone ethylene, which is involved in ester synthesis, as demonstrated during apple or banana storage in the presence of its antagonist 1-MCP [155,156,157].

### 5.2. Terpenoid Volatiles

Several studies in *Citrus* have highlighted important changes in terpenoid volatiles monitored by HS-SPME–GC–MS. These changes were related to CI and tolerance to cold storage. In mandarins, the accumulation of terpenoid volatiles is associated with chilling-sensitive fruits [158]. This increase is temperature dependent, and the authors suggest that it was responsible for decreased fruit palatability, as terpenes can contribute to an unpleasant aroma, providing ‘musty’, ‘resinous’ and ‘oily’ notes.

Another study on volatile emission by intact grapefruits stored at 12 and 2 °C for 7 weeks outlined important differences in the profiles of the terpenoid volatiles as a consequence of temperature [98]. Interestingly, grapefruits stored at 2 °C experienced a strong increase in monoterpene content, particularly in limonene and β-myrcene levels; this group of volatiles was strongly decreased in fruits stored at 12 °C at the beginning of the postharvest period, after which their content remained unchanged. In contrast, sesquiterpene emissions were predominant in the fruits stored at 12 °C [98]. While the accumulation of the monoterpene β-myrcene under 2 °C can negatively impact negatively consumer acceptance, by providing ‘musty’ and ‘wet soil’ aroma notes to the fruits [108], sesquiterpene ketone nootkatone levels, an important volatile in grapefruit aroma, seems to be promoted (in term of it content) under moderate–intermediate-temperature storage, but not refrigerated conditions [98,159] (Table 3). Taken together, these data suggest that the aroma quality of grapefruits could be better maintained during intermediate-temperature storage, as has been previously demonstrated in mandarins [126,149].

The trend in the accumulation of the monoterpene limonene and the sesquiterpene α-farnesene, which showed a transient increase after one week of storage under the two different temperatures, could be related to cold-induced responses. Limonene release, measured both by HS-SPME–GC–MS and capillary gas–liquid chromatography–MS, has also been described in other *Citrus* species [125,126,127,160] and could be a consequence of cell wall and plasmatic membrane disruptions in the oil glands [99,125]. The degree of accumulation of α-farnesene is correlated with the susceptibility of different cold-sensitive *Citrus* species to CI development at 0 °C storage [128]. Interestingly, α-farnesene stopped being emitted by grapefruits after 3 weeks of storage at 12 °C, which coincided with the decrease in the observed CI symptoms, i.e., peel injury. At 2 °C, α-farnesene emissions were maintained during the whole postharvest period, concomitant with the CI symptom progression, confirming the relation between the detection of this volatile and CI manifestation. In this sense, the detection of α-farnesene by metabolome-driven approaches could be of high value as a potential biomarker to assess *Citrus* quality during postharvest (Table 3). Similarly, the monoterpene linalool, a key component of the aroma of papaya, was negatively affected by cold postharvest storage at 10 °C, and a concomitant downregulation of linalool synthase expression was also observed, suggesting that this volatile could be used as a marker to define papaya quality during postharvest storage [129] (Table 3). Linalool is also the predominant compound responsible for flavor in Muscat table grapes, a highly appreciated quality trait [161]. The postharvest storage of ‘Shine Muscat’ grapes at different temperatures between 0 and 10 °C showed that the decrease in linalool, profiled by HS-SPME–GC–MS, was enhanced at relatively low (0, 2 and 5 °C) temperatures in both fruit skin and flesh. Concomitant with linalool levels, grapes stored for four weeks at 10 °C presented a higher Muscat flavor than grapes stored at 0 °C for the same duration [130]. A possible effect of temperature on linalool synthesis or on the interconversion of free (aroma-producing) linalool and its glycosidically (odorless) bound form could be responsible for the observed differences in its concentration. In this sense, optimal storage at 10 °C for a short period or at relatively low temperatures followed by poststorage conditioning at 10 °C is fundamental for maintaining aroma quality for consumers [130] (Table 3).

The combination of 1-MCP with high O_2_ or high CO_2_ seemed to favor terpene content, as did the CO_2_-enriched atmosphere in lemon [162]. CA storage under an elevated CO_2_ atmosphere (8% CO_2_ and 2%–3% O_2_) could also promote terpene accumulation in mango. Indeed, fruit injury as a result of CA can enhance the activity of glycosidases, releasing monoterpenes, such as linalool or terpineol, from their glycoside-bound forms [163]. However, all tested CA treatments resulted in a reduction in total sesquiterpenes and also enhanced levels of ethanol, acetaldehyde and esters compared to those under atmospheric conditions. In addition, it was established that mangoes should not be stored under 3%–5% O_2_ to avoid excess fermentative compound accumulation and maintain fruit aroma quality [163,164].

## 6. Conclusions

Although fruit responses to postharvest storage conditions are species and even cultivar dependent, making them especially complicated to study, metabolomic approaches alone or combined with transcriptomic/proteomic analyses are highly useful for understanding how metabolic changes affect quality traits. In particular, reconfiguration of fruit metabolism as a consequence of the abiotic/biotic stress encountered during postharvest storage conditions (cold, hypoxia, pathogens, etc.) has a direct impact on the accumulation of taste- and aroma-producing metabolites, which are decisive attributes for consumers and thus for the fruit industry. Even though many molecular mechanisms active during fruit postharvest storage and senescence remain elusive, future omic studies will shed light on them to optimize fruit storage conditions.

Furthermore, the recent advances in metabolomic-driven technology allows the identification of valuable biomarkers that can be employed by the fruit industry to tightly monitor changes in quality attributes during postharvest storage. In this sense, the use of multiplatform approaches offers the possibility to select a set of metabolite markers, which could better depict the impact of postharvest storage on aroma, taste, appearance and nutritional value [165].

## Figures and Tables

**Table 1:** Primary metabolites (sugars, organic and amino acids) identified as putative biomarkers by metabolomic profiling studies to assess fruit quality changes during postharvest storage. d: day; RT: room temperature; HPLC: high-performance liquid chromatography; UHPLC–MS/MS: ultra-high-pressure liquid chromatography–tandem mass spectrometry; ^1^H-NMR: proton-NMR; CI: chilling injury; ROS: reactive oxygen species.

Metabolite	Effect on Fruit	Postharvest Treatment	Behavior during Postharvest	Fruit Species	Metabolomic Platform	Reference
raffinose, galactinol	Tolerance to CI	39 °C, 3 d + 0 °C, 2 d	Increase	peach	GC–MS	[52]
raffinose, galactinol, myo-inositol, trehalose	Enhanced capacity to cope with postharvest stress conditions	20 °C, 14 d	Increase	non-climacteric plum	NMR, UHPLC–MS/MS	[46]
malate	Decrease in water loss and in susceptibility to opportunistic fungal infections	RT, 20 d	Decrease	tomato	GC–MS	[57]
malate	Correlation with fruit firmness and shelf-life	25 °C until first symptoms of deterioration	Decrease	tomato	GC–MS, ^1^H-NMR	[58]
mannose, citramalate, gluconate, keto-gulonate		18 °C, 10 d	Increase	tomato	GC–MS	[36,59]
dopamine	Conversion to salsolinol at late postharvest stages, decrease in fruit quality	25 °C until senescence	Decrease	banana	^1^H-NMR	[36]
proline	Osmoprotection and ROS-scavenging functions	0 °C, 20 kPa CO_2_/20 kPa O_2_/60 kPa N_2_, 3 d + 0 °C, air, 30 d + 20 °C, 2 d	Increase	grape	LC–MS	[60]
proline	Osmoprotection and ROS-scavenging functions	1 mM GABA treatment, 20 min + 4 °C, 18 d (dark)	Increase	zucchini	HPLC	[61]

**Table 2:** Secondary metabolites (polyphenols and carotenoids) identified as putative biomarkers by metabolomic profiling studies to assess fruit quality changes during postharvest storage. d: day; w: weeks; UHPLC–HRAM MS^n^: ultra-high-performance liquid chromatography coupled to high resolution multiple-stage mass spectrometry.

Metabolite	Effect on Fruit	Postharvest Treatment	Behavior during Postharvest	Fruit Species	Metabolomic Platform	Reference
taxifolin deoxyhexoside, taxifolin hexoside tetrahydroxyflavanone-*O*-deoxyhexoside	Antifungal activity, withering stress responses	withering, 91 d	Increase	grape	Untargeted HPLC–MS	[86]
procyanidin B1, epi-catechin	Resistance to *Penicillium expansum*	2 °C storage	Increase	apple	UHPLC–HRAM MS^n^	[94]
β-cryptoxanthin	Part of β, β-xanthophyll pool in mature oranges	12 °C up to 7 w	Increase	sweet orange	HPLC	[96]

**Table 3:** Volatile compounds identified as putative biomarkers to evaluate the effects of postharvest storage on fruit aroma. d: day; w: week; GC–O: gas chromatography–olfactometry; GLC–MS: capillary gas–liquid chromatography–mass spectrometry.

Volatile	Effect on Fruit	Postharvest Treatment	Behavior during Postharvest	Fruit Species	Metabolomic Platform	Reference
ethanol, ethyl acetate, acetaldehyde	‘Off-aroma’ generation, ‘alcohol’ aroma	3 °C, 3 ws supplemented with different CO_2_ concentration	Increase	strawberry	HS-SPME–GC–MS	[117]
ethanol, ethyl acetate, acetaldehyde	‘Off-aroma’ generation, ‘alcohol’ aroma	5 °C, 6 ws + 20 °C, 1 w	Increase	mandarin	HS-SPME–GC–MS and GC–O	[109]
ethanol, ethyl acetate, acetaldehyde	‘Off-aroma’ generation, ‘alcohol’ aroma	2.5 °C, 7 d + 1 °C followed by two different low oxygen protocols up to 240 d	Increase	apple	HS-SPME–GC–MS	[11]
ethanol, ethyl acetate, acetaldehyde	‘Off-aroma’ generation, ‘alcohol’ aroma	0 °C, 6 w + 20 °C, 2 days supplemented with different CO_2_ concentration	Increase	grape	HS-SPME–GC–MS	[87]
β-myrcene	Decrease in aroma quality	2 °C or 12 °C, 7 w	Increase at 2 °C, decrease at 12 °C	grapefruit	HS-SPME–GC–MS	[98]
ketone nootkatone	Confers characteristic ripe aroma fragrance	12 °C, 7 w	Increase in 12 °C	grapefruit	HS-SPME–GC–MS	[98]
limonene	Cold-induced responses	2 °C, 7 w	Increase	grapefruit	HS-SPME–GC–MS	[98]
limonene	Cold-induced responses	1 °C, 7 w	Increase	lemon	HS-SPME–GC–MS	[125]
limonene	Cold-induced responses	5 °C, up to 6 w	Increase	mandarin	HS-SPME–GC–MS	[126]
limonene	Senescence predictor	Combination of treatments, including 15 °C, 7 d + 2 °C, 18 d, 13 °C, 17 d	Increase	grapefruit	GLC–MS	[127]
α-farnesene	Correlation with CI symptom development in 0 °C storage	0 °C up to 12 w, with or without ethylene	Increase	lime, mandarin, grapefruit, orange	GC–MS	[128]
linalool	Key component of fruit aroma	10 °C, 10 d + 22 °C until fully ripe	Decrease in low-temperature storage	papaya	HS-SPME–GC–MS	[129]
linalool	Key component of fruit aroma	0, 2, 5, and 10 °C up to 3 months	Decrease in low-temperature storage	Muscat table grapes	HS-SPME–GC–MS	[130]
